# Clinical trials to go green–A sustainable argument for decentralised digital clinical trials

**DOI:** 10.1371/journal.pdig.0000366

**Published:** 2023-10-24

**Authors:** Simon H. Kohl, Caroline Schmidt-Lucke

**Affiliations:** 1 MEDIACC GmbH, Sächsische Str. 70, 10707 Berlin, Germany; 2 Charité-Universitätsmedizin Berlin, Berlin, Germany; Iran University of Medical Sciences, IRAN (ISLAMIC REPUBLIC OF)

The digital revolution has made its way into the highly regulated field of clinical trials. With an increase in the use of digital technologies in clinical trials decentralised digital clinical trials (DDCTs) are disrupting the way evidence is generated for new medical treatments [1,2]. Recently, the European Union and Switzerland have published the first guidelines for DDCTs, providing a starting point for key stakeholders to understand the process and requirements [3,4]. Despite known advantages of DDCTs over traditional at least in part paper-based trials, there is still reluctance to the seemingly technically more complicated DDCTs. DDCTs are, however, faster and more cost-effective, more accessible for patients with the possibility to participate independently from where they live or their socio-economic background and reduce documentation effort. This improves recruitment rates and leads to more diversity in the representative study population enabling a better basis for decision-making in the health care system [5,6].

Since climate warming poses a multifaceted threat, impacting the environment and economy [7,8], it is worth focusing on another important previously unexplored aspect of the digitisation of clinical trials: saving energy and CO_2_ emissions.

Informed consent forms, data protection information, test forms, questionnaires, registration forms, and visits to the study centres consume a lot of paper and energy in traditional clinical trials. Hence, digitising clinical trials not only improves the clinical trial system, but is also less harmful for the environment. But how much do we really save by fully digitising a clinical trial?

The aim of this opinion article was to add an additional and moreover, the most sustainable argument for DDCTs, namely the large reduction of energy and CO_2_ emissions of DDCTs compared to traditional trials. To address this topic, we estimated the CO_2_ savings of various scenarios involving decentralised and digital elements in comparison to a traditional middle-sized clinical trial from our institution.

Assuming a 25% dropout rate, a typical clinical trial with 2000 patients visiting one of the ten study sites nine times would require a total of 164,800 sheets of paper for case reports and patient forms, the study master file, and site files. This corresponds to a stack of paper higher than 16m and 799 kg CO_2_ [9]. Additionally, monitoring visits to nationally distributed study centres are commonly necessary. On average, each of the ten centres will be monitored five times throughout the course of a clinical trial. If we assume the clinical monitor will be travelling to each of the centers by car, another 3,808 kg of CO_2_ would be emitted into the atmosphere as estimated using the model by [10].

Further, when study participants are required to travel to the study centres, an additional 237 kg of CO_2_ will be emitted into the atmosphere. Finally, study documents need to be sent back and forth between the monitor and the centres over the course of the study. With one letter per patient and 20 g of CO_2_ per letter [11], this adds up to another 40 kg of CO_2_. In total, this exemplary traditional, paper-based, clinical trial produced 4,885 kg of CO_2_.

Of course, DDCTs also need energy. Reading documents, filling out questionnaires on computers or smartphones (1 minute per page) [12], video consultations (1 hour with each study centre) [13], emails [11] (12 per patient), and–the largest part—server times (assuming 0.04 kw/h) to run and operate the study platform for two years all need electricity and emit CO_2_. In our exemplary study above, however, this amounts to just 486 kg CO_2_. Noteworthy, even this seemingly small proportion of CO_2_ emission should make us aware of our responsibility and drive us to formulate precise scientific questions, which are then implemented consistently with high data quality in order to achieve meaningful results.

Hence, a classical clinical trial needs about 10 times more CO_2_ and the full digitisation of a clinical trial saves 4,399 kg (90.1% reduction) of CO_2_. That is equivalent to a long-distance flight of 24,000 km [10] (almost four times from Berlin to New York) and 237 trees would have to grow for one year to make up for it [14]. Clearly, full digitisation of a clinical trial remains an ambitious goal and is not applicable in many study scenarios. The digitisation of clinical trial elements can pose additional risks and ethical concerns, especially when dealing with very vulnerable populations or complex medical procedures that require face-to-face interactions with physicians. Also, individuals who have limited access to digital tools or are not comfortable using them and may be excluded from a fully digitised trial.

As regulations rightfully stipulate, the rights, safety, dignity, and well-being of study participants should always be prioritised. Any additional burden placed on participants must be carefully weighed against the benefits of digitization [4]. Therefore, other scenarios that involve different levels of decentralised and digitised practices (hybrid studies) offer a more realistic solution while still significantly reducing carbon emissions.

If we extrapolate the estimations for the three different scenarios to the approximately 15,000 clinical trials starting every year in Europe, according to the International Clinical Trial Registry Platform of the WHO [15], there is the potential to save between 41,009 t and 65,981 t CO_2_ per year (see Fig 1). The European Climate Law sets a clear target of achieving climate neutrality by 2050. The law mandates that all EU policies align with this objective and that all sectors of the economy and society–including medical research play their part [16]. By embracing DDCTs regulatory authorities, policymakers, funders, health care companies and researchers can play their part in achieving these legally mandated climate targets. It is about time to for all interested parties to sit down at the same table to securely enable this aim.

**Fig 1:**
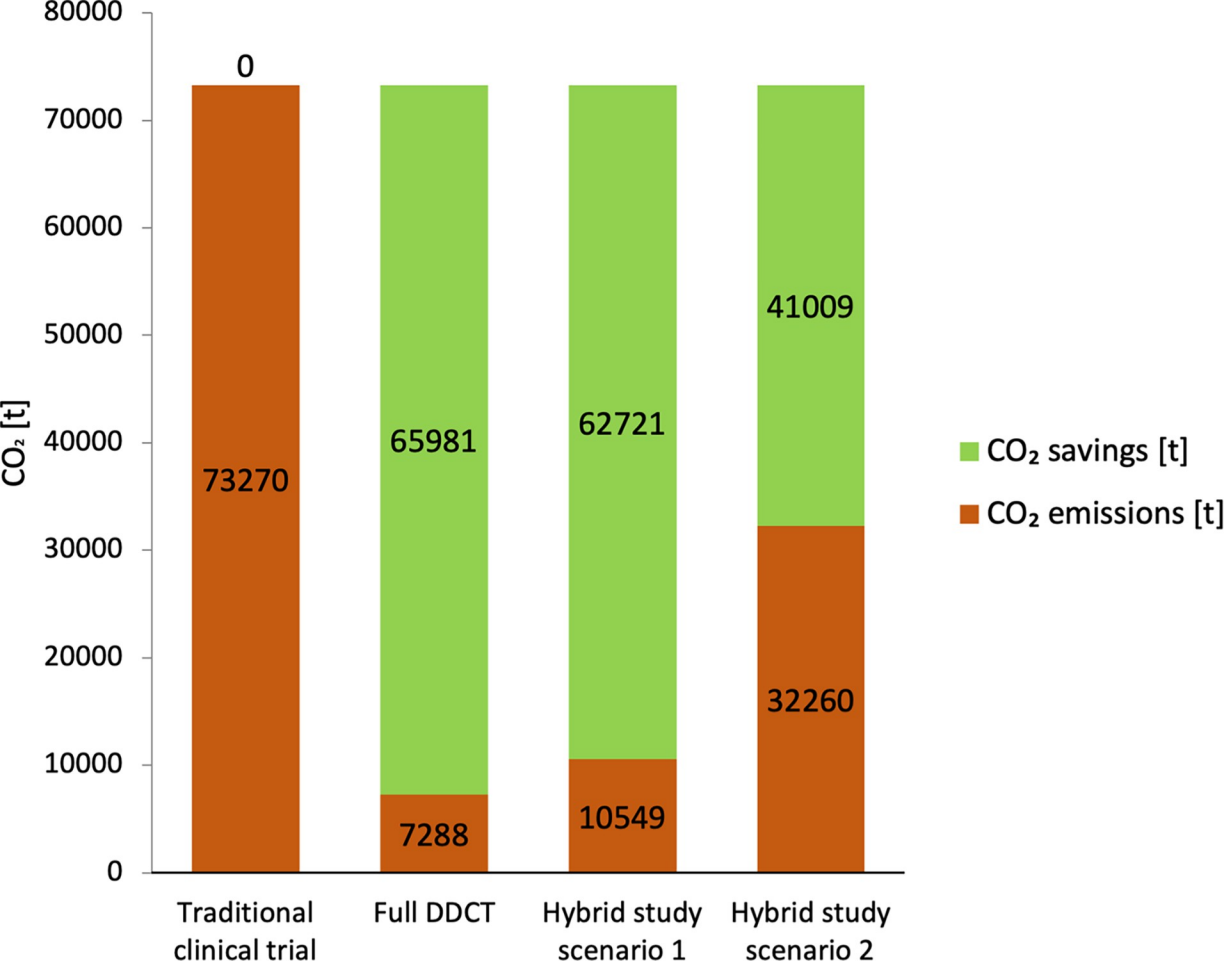
Estimated CO_2_ emissions and potential savings per year through decentralised and digital clinical trial (DDCT) methods in Europe. The figure illustrates the estimated CO_2_ emissions (brown columns) and potential savings (green columns) in different study scenarios comparing traditional clinical trial methods with various degrees of adoption of DDCT methods. The analysis focuses on the approximately 15,000 clinical trials initiated annually in Europe [15]. Full DDCT: fully digitized and decentralized trial without any study centres or paper-based processes; hybrid study scenario 1: digital clinical trial (DCT) including visits of the participants to the study centres but no on-site monitoring; hybrid study scenario 2: DCT including only an initiation and close-out visit, but no on-site monitoring visits in between.

## Data availability statement

This is not an original research article. A spread sheet including our CO_2_ estimations and assumptions can be found in S1 File.

## Supporting information

S1 File CO_2_ estimations and assumptions for three different study scenarios. (XLSX)
